# Minimizing moving distance in deposition behavior of the subterranean termite

**DOI:** 10.1002/ece3.6051

**Published:** 2020-01-29

**Authors:** Sang‐Bin Lee, Nan‐Yao Su, Hark‐Soo Song, Sang‐Hee Lee

**Affiliations:** ^1^ Department of Entomology and Nematology Ft. Lauderdale Research and Education Center Institute of Food and Agricultural Sciences University of Florida Ft. Lauderdale FL USA; ^2^ Division of Industrial Mathematics National Institute for Mathematical Sciences Daejeon Korea

**Keywords:** *Coptotermes formosanus*, foraging efficiency, Formosan subterranean termite, social insect, tunneling

## Abstract

Subterranean termite nests are located underground and termites forage out by constructing tunnels to reach food resources, and tunneling behavior is critical in order to maximize the foraging efficiency. Excavation, transportation, and deposition behavior are involved in the tunneling, and termites have to move back and forth to do this. Although there are three sequential behaviors, excavation has been the focus of most previous studies. In this study, we investigated the deposition behavior of the Formosan subterranean termite, *Coptotermes formosanus* Shiraki, in experimental arenas having different widths (2, 3, and 4 mm), and characterized the function of deposited particles. We also simulated moving distance of the termites in different functions. Our results showed that total amounts of deposited particles were significantly higher in broad (4 mm width) than narrow (2 mm) tunnels and most deposited particles were observed near the tip of the tunnel regardless of tunnel widths. In addition, we found that deposited particles followed a quadratic decrease function, and simulation results showed that moving distance of termites in this function was the shortest. The quadratic decrease function of deposited particles in both experiment and simulation suggested that short moving distance in the decrease quadratic function is a strategy to minimize moving distance during the deposition behavior.

## Graphical abstract

Subterranean termites nest underground and forage out by constructing a network of tunnel. Tunneling behavior of the termites is composed of excavation, transportation, and deposition, and little is known for deposition behavior. We found that deposited particle distribution in the tunnel followed a quadratic decrease function in the experiment. In addition to the experiment, the simulation results revealed the shortest moving distance was observed in the quadratic decrease function.

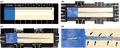

## 1 INTRODUCTION

Foraging is one of the most important factors affecting life history and fitness. Foraging behavior is often considered as a collective behavior, especially in social insects, as integrated actions of individuals. In social insects, such as subterranean termites and ants, a colony may contain hundreds of thousands to millions of individuals. Many species of ants excavate underground nests using subterranean networks to connect chambers for food storage, brood rearing, etc (Brian, 2012; Délye, 1971; Gautrais, Buhl, Valverde, Kuntz, & Theraulaz, 2014; Hölldobler & Wilson, 1990; Tschinkel, 2003). For these ant colonies, the tunnel connects the underground nest to soil surface. Similar to ants, the central nest of subterranean termites is located below the ground, but termites construct extensive underground tunnels to search for food and to transport acquired food to take to the central nest. Therefore, construction of tunnels that maximize both food search and food transportation efficiency is a key to their ecological and evolutionary success.

The underground tunnels built, for instance, by *Coptotermes formosanus* Shiraki, and extend up to 100 m (King & Spink, 1969; Su & Scheffrahn, 1988). Consequently, for subterranean termites, tunnel geometry is a key component to maximize foraging efficiency, which is composed of searching and transportation of food (Lee, Bardunias, & Su, 2006, 2007; Lee & Su, 2009). Tunnel distribution of *Retculitermes flavipes* (Kollar) termites optimized food searching efficiency (Robson et al., 1995), and tunnel branch length distribution of *C. formosanus* termites optimized searching and transporting (Lee, Bardunias, & Su, 2007).

Despite of the importance, however, investigating the overall tunnel structure for subterranean termite colonies in the field is labor‐intensive, time‐consuming, and costly. To overcome this problem, termite tunnel patterns were studied in small scale planar arenas (0.1–1.0 m) in the laboratory (Campora & Grace, 2001; Chouvenc, Bardunias, Li, Elliott, & Su, 2011; Su, Stith, Puche, & Bardunias, 2004). These experiments enabled us to investigate the geometry of tunnels as well as tunneling behavior of subterranean termites with less disturbance and greater visibility. Tunneling behavior defines the pattern of tunnels formed by termites. Thus, a better understating of this behavior could help to define the ecological significance of tunneling patterns. Accordingly, numerous studies have investigated tunneling behavior of subterranean termites using the planar arena, such as tunnel geometry (Puche & Su, 2001; Su et al., 2004), tunnel orientation (Campora & Grace, 2001), task allocation during excavation (Cornelius, 2012; Cornelius & Gallatin, 2015; Yang, Su, & Bardunias, 2009), behavioral responses (Lee, Bardunias, & Su, 2008c; Lee Yang, & Su, 2008a, 2008b) sand displacement (Li & Su, 2008, 2009), branch formation (Hedlund & Henderson, 1999; Robson et al., 1995), and tunnel volume regulation (Bardunias & Su, 2009, 2010; Su & Lee, 2009).

These studies have deepened our understanding of the relationship between termite tunnel patterns and foraging efficiency, but there is still a lack of detailed research into the process of soil excavation–deposition at the individual termite level (Li & Su, 2009). It has been speculated that spaces of underground galleries of subterranean termites were created by compacting soil (Ebeling & Pence, 1957; Greaves, 1962; Greaves & Florence, 1966; King & Spink, 1969) may create tunnels by compacting soil. This speculation, however, was rejected by Li and Su (2008), who revealed that subterranean termites removed sand particles from excavation sites to deposition sites during the tunnel excavation process. This mechanism is similar to other social insects (i.e., ants), in which workers carried a soil pellet using their mandibles to the surface and deposited in a certain area (Sudd, 1969). However, there is no such surface in subterranean termites and they have to excavate and deposit along the tunnel. Li and Su (2008) proposed the wood consumption hypothesis, which states that excavated sand is deposited in the void space created by consuming wood. Further, Brown, Broussard, Kard, Smith, and Smith (2008) reported a significant correlation between wood consumption and the amount of soil excavated.

Tunnel geometry such as length and width of subterranean termites is highly variable (Grace, Aihara‐Sasaki, & Yates, 2004; Hedlund & Henderson, 1999), and tunneling behaviors are affected by both internal and external factors such as genetical, physiological, and environmental conditions (Lee, Yang, & Su, 2008b). Tunnel widths formed by *C. formosanus* ranged from 2 to 5 mm (Campora & Grace, 2004; Hapukotuwa & Grace, 2012) or even much wider (i.e., 1.7–3.4 cm; Bardunias & Su, 2010; Puche & Su, 2001). The widths was related to either worker body size, implying that larger workers are better excavators because of their larger mouth parts than smaller workers (Campora & Grace, 2004; Crosland, Lok, Wong, Shakarad, & Traniello, 1997) and queue size (Bardunias & Su, 2010).

Tunnel width is an important factor because it critically affects traffic efficiency of subterranean termites. In narrower tunnels (i.e., 2 mm), termites showed three different types of behavior: (a) turn and pass through, (b) one individual change directions (e.g., turn back), and (c) stop and start excavating another tunnel once there is a queue (Sim & Lee, 2012). In 3 mm of tunnel width, termites touched the tunnel wall with their antennae and walk through undisturbed. In 4 mm of tunnel width, the tunnel was wide enough for one individual to pass through. Regardless of other individuals in the tunnel, they can pass through without traffic jam. However, walking speed is decreased because it takes more time to touch both tunnel walls.

In addition, opposing headings in excavation and deposition facilitate branch formation in tunnels (Bardunias & Su, 2009). This branch formation, branch tunnel length distribution, was determined as the exponentially decaying function in the empirical study, and a simulation result showed that the ratio of energy gain from food acquisition to loss of food transporting for a given time was maximized at 0.15–0.20, which is similar to empirical tunnel patterns (Lee et al., 2007).

Although two different tunneling behavior components are known in construction of tunnels, the excavation behavior has received more attention than deposition behavior. Consequently, relatively little is known of the deposition behavior of subterranean termites, and the amount of deposition particles has never been quantified. We hypothesized that termites may deposit more particles near the tips of tunnels than along the lengths of the tunnels and that this may be related to transportation efficiency in the tunnel.

To test the hypothesis, we first measured the amount of deposited particles in different tunnel widths using image processing tools and determined the mathematical function of deposition distribution. Based on the results of deposited particle distribution, we further simulated the moving distance of termites in different functions of its distribution to examine the moving distance in relation to sand particle deposition pattern. In this study, we focused on finding the deposited sand distribution in the tunnels of subterranean termites and discussed what this distribution means in relation to the foraging efficiency.

## 2 MATERIALS AND METHODS

### 2.1 Termites

Termites were collected from three different colonies in Broward County in Florida, USA, using bucket traps, (Su & Scheffrahn, 1986). Collected termites were separated from the debris and introduced into a covered box (30.5 × 45.7 × 15.2 cm, Carlisle, Oklahoma City, OK, USA) containing organic soil at the bottom (8 cm depth) with 10 wooden blocks of *Picea* sp. on top. The container was moistened using deionized water every week, and the temperature was kept at 28 ± 1°C.

### 2.2 Sand deposition experiments

**Figure 1:**
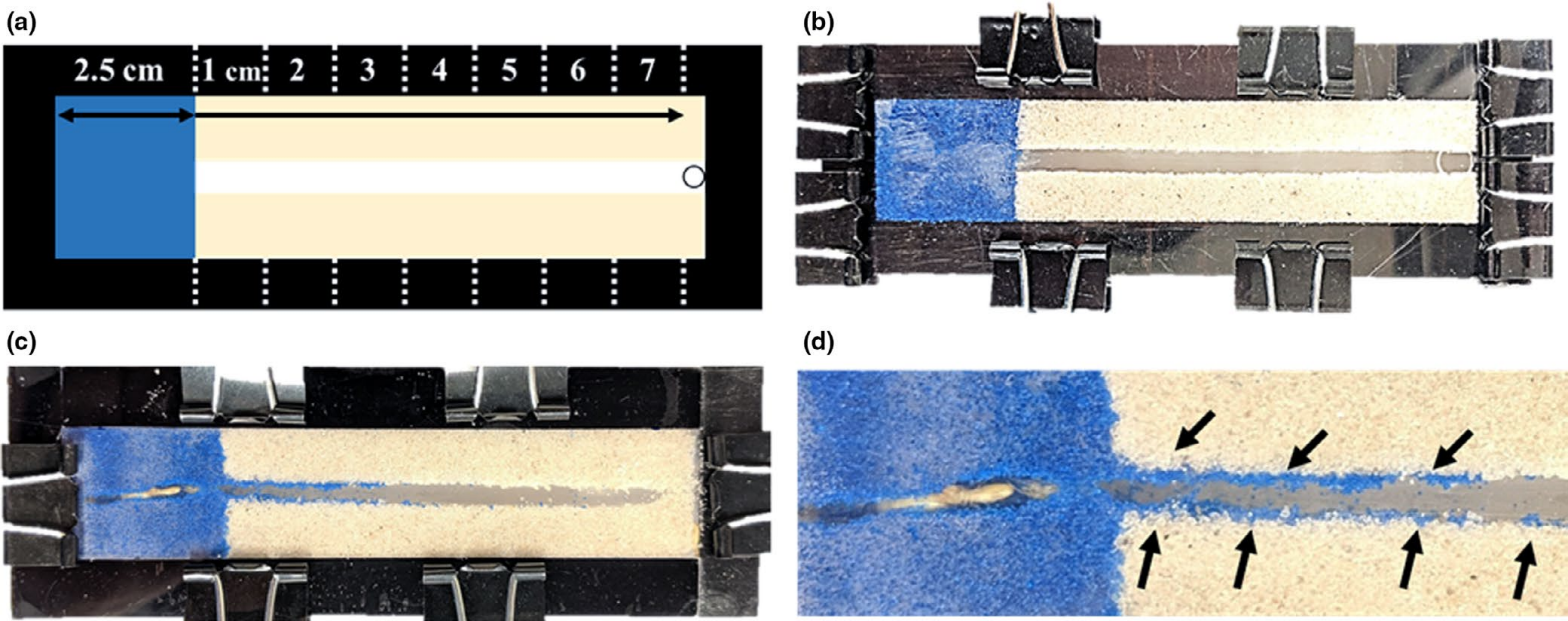
An experimental arena is composed of three layers of acryl, blue sand (left side), yellow sand with preformed tunnel, and entrance hole (circle on the right). Experimental arena design (a). An example of an arena before (b), and after (c) the experiment. Deposited sand particles in an enlarged view (d). Black arrows in d) indicate deposited sand particles after excavation in the blue sand area

The experimental arena was made of three layers of acryl, in which the top and bottom layers acted as a frame, and the middle layer created a space (Figure 1). Blue and yellow sand (0.3 ~ 0.7 mm, sieved) were moistened with deionized water (≈7% by sand weight). The arena was filled with blue sand (left side) from the edge to 2.5 cm, and the rest of the space was filled by yellow sand (Figure 1b). In order to create preformed tunnels in the yellow sand, templates of different widths (2, 3, and 4 mm) were used. Sand was compacted using hand rollers. After compacting the sand, four workers that had similar body sizes were randomly chosen and introduced through an entrance hole (diameter: 5 mm) on the top layer (right side) (Figure 1a). Experiments were carried out for four hours and terminated when termites tunneled through entire 2.5 cm of blue sand. We excluded samples if the excavated tunnels did tunnel through blue sand or if the shape of the excavated tunnels was bent. Only samples in which the tunnels were straight and reached the end of the arena were selected for further analysis. This was done to ensure the amount of excavated sand was similar in all experiments.

### 2.3 Data measurement and sand particle counting

Once the experiments were completed, the arena was placed into the freezer for ten min to stop termite activity and dried in the oven at 70°C for three hours to extract the sand particles. Deposited sand particles were collected at one‐cm intervals from the border between the blue and yellow sand to the introduction hole (Figure 1a). Collected sand was spread out on white paper, and photographs were taken using a digital camera (Sony α 5100, Tokyo, Japan) under LED light.

To count the number of blue sand particles, the color image was first converted to a grayscale image using the rgb2gray () function provided by MATLAB R2016b (MathWork Inc). We set the threshold value on the gray image. For each pixel in the image greater than the threshold value, we assigned a value of one to the pixel, and a value of zero to each pixel with a value less than the threshold. The threshold value was set appropriately at a level sufficient to visually distinguish between blue and yellow sand particles. Through this process, we changed the original color image to a binary image. A chunk of pixels with a value of one can be understood as one blue sand particle. In this image analysis, we calculated the number and size of pixel. For this calculation, we programed a MATLAB function that checks which values the neighbors surrounding a pixel with a value of one have. This function starts at one pixel and examines all the pixels connected to it, and, if the neighboring pixels all have a value of zero, it returns the size value of the chunk corresponding to one sand particle. We performed this process for every pixel with a value of one in the image. We eventually eliminated the case where the size of each chunk was too small, defined 0.3 times as small as average, or too large, defined 2.5 times as large as average. Chunks that were too small were mostly fragments of broken sand particles, and chunks that were too large were shadows created by reflected light.

### 2.4 Simulation model for moving distance

To investigate which distribution function is most efficient in regard to moving distance, we calculated the energy consumption for four types of distribution functions (quadratic decreasing, quadratic increasing, constant, and Gaussian functions). To calculate the moving distance, we performed a simple computer simulation. We set two points A and B, 50 cm away from each other, in the simulation space. Then, we placed 100 simulated sand particles in site A and had the termites move all the sand to site B. Simulated termites were programmed to move one sand particle at a time toward site B. The distance the sand was moved was determined by the distribution function. A simulated termite picked up a sand particle at site A and dropped the sand as it walked toward site B. The other simulated termites at the back picked up the dropped sand again and dropped it closer to site B. Through repetition of this process, we calculated how many times the termites moved, on average, when all the sand particles were moved to site B. We repeated this 3,000 times for each distribution function of sand. Counting sand particles and simulations were conducted under MATLAB R2016b (MathWork Inc).

### 2.5 Statistical analyses

A total of three colonies of *C. formosanus* were examined with the combination of five replications for 2, 3, and 4 mm widths, yielding 15 replications for each width (three colonies × five replicates). The total numbers of deposited sand particles were calculated and were subjected to a one‐way analysis of variance (ANOVA; α = 0.05) with width as a factor and Tukey's honestly significant difference (HSD) test as a post hoc analysis to discern statistically different groups.

In addition, the average numbers of deposited sand particles in different widths across distances were analyzed using a two‐way ANOVA (α = 0.05) with widths and distances as the factors. Also, Tukey's HSD was subjected to discern statistically different groups.

Furthermore, the curve of the average numbers of deposited sand particles by distance was fitted into different models, that is, linear, logarithmic, quadratic, compound, power, exponential, and logistic, in order to determine which models(s) were best suited for explaining the number of deposition particles.

The average moving distances from the results of the simulations were compared among functions (quadratic decrease, constant, quadratic increase, and Gaussian functions), with the Kruskal–Wallis test and pairwise multiple comparison test as post hoc analyses. All statistical analyses were carried out in SPSS version 19.0 (IBM SPSS Inc., 2016).

### 2.6 Ethical note

This study was primarily focused on observation of behavior. Neither specific permits nor licenses were required for the collection of termites from the field and for maintenance in laboratory. Also, no endangered or protected species were used in this study.

## 3 RESULTS

**Figure 2:**
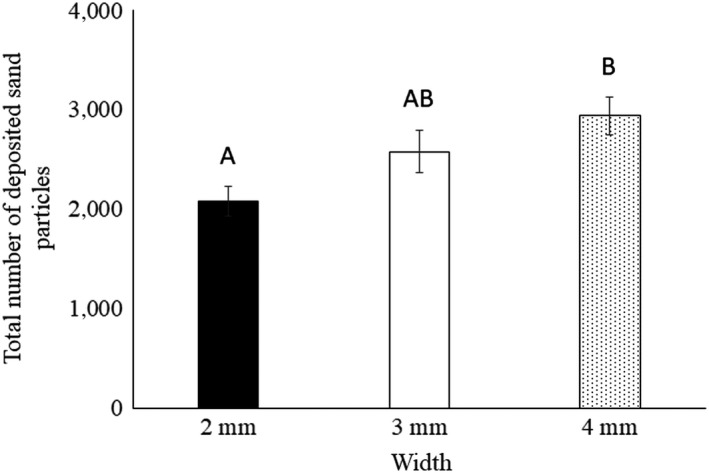
Average of total number of deposited sand particles by four workers of C. formosanus. Different uppercase letters denote statistically significant differences according to Tukey's HSD (α = 0.05). Black, white, and dotted bars are 2, 3, and 4 mm, respectively

During the experiments, termites kept moving back and forth to excavate, transport, and deposit the blue sand particles. Excavated sand particles (blue sand) were deposited on both sides of the tunnel walls. The averages of the total number of deposited sand particles were significantly different among width (*F*_2,42_ = 4.92, *p* = .012). The number of deposited sand particles was the highest in the 4 mm tunnel width and the lowest in the 2 mm width (Figure 2).

**Figure 3:**
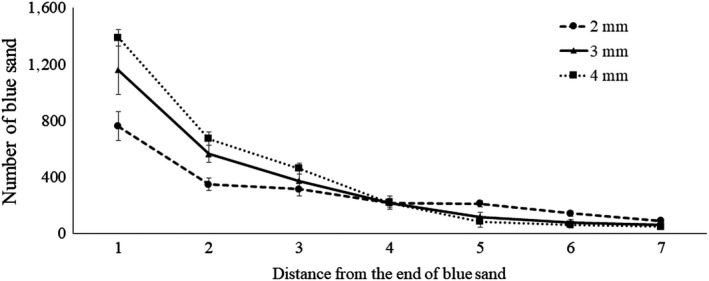
Distributed pattern of average number of deposited sand particles by four workers of C.formosanus. Circle, triangle, and square markers are 2, 3, and 4 mm widths, respectively

The average numbers of deposited sand particles were significantly different among width (*F*_2, 313_ = 7.49, *p* = .001), distance (*F*_6, 313_ = 114.77, *p* < .0001), and in its interactions (*F*_12, 213_ = 5.40, *p* < .0001; Table 1). Overall, termites deposited significantly more sand particles near the end of the preformed tunnel that borders the blue sand, and the numbers of deposited sand particles significantly decreased as distances increased from the blue sand area (Table 1, Figure 3).

**Table 1:** Average (mean ± *SEM*) numbers of deposited sand particles (blue sand) by four workers of *C. formosanus* in different tunnel widths and distances from the border of blue and yellow sand

Distances/width (cm)	2 mm	3 mm	4 mm
1	761.1 ± 56.6 Ae	1,160.7 ± 171.4 Be	1,388.8 ± 103.9 Be
2	347.4 ± 47.1 Ad	565.3 ± 62.3 Bd	672.0 ± 43.8 Bd
3	314.9 ± 36.8 Ac	373.3 ± 69.2 Bc	461.1 ± 47.2 Bc
4	218.3 ± 24.6 Ab	218.7 ± 32.1 Bb	219.3 ± 45.8 Bb
5	211.5 ± 39.7 Aab	118.5 ± 30.8 Bab	85.3 ± 21.2 Bab
6	142.7 ± 22.5 Aab	80.4 ± 21.6 Bab	64.3 ± 19.8 Bab
7	89.5 ± 23.4 Aa	63.2 ± 14.6 Ba	49.4 ± 13.1 Ba

Means followed by different uppercase letters within a row and different lowercase letters within a column denote significant differences according to Tukey's HSD (*α* = 0.05).

Among tested functions for curve fitting, the logarithmic function fits well for the 2 mm width (*F* = 163.56; *R* ^2^ = 0.61; *p* < .0001), and the quadratic function fits well for the 3 mm (*F* = 71.40; *R* ^2^ = 0.583; *p* < .0001) and 4 mm (*F* = 230.00; *R* ^2^ = 0.82; *p* < .0001) widths (Figure 3). If data were pooled together regardless of width, the highest R‐square values were observed in the quadratic functions (*F* = 59.34; *R* ^2^ = 0.54; *p* < .0001; Figure 3).

**Figure 4:**
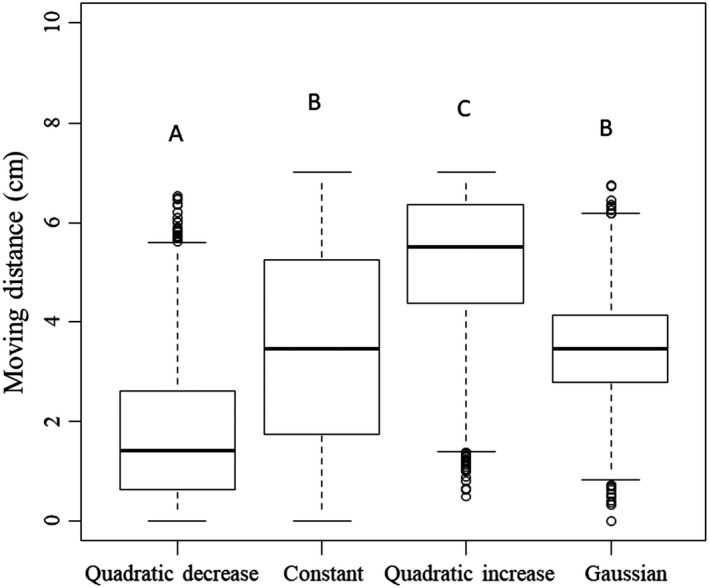
Simulation results of average moving distance depending on distribution functions (quadratic decrease, constant, quadratic increase, and Gaussian). Thick lines in the boxes indicate the mean, and circles above and below the box are outliers. Different uppercase letters in the figure denote significant differences according to a pairwise comparison (α = 0.05)

In the simulation result, there were significant differences between moving distances depending on distribution functions (Kruskal–Wallis H_3, 2998_:4,795.66, *p* < .0001; Figure 4). The shortest moving distance was observed in the quadratic decrease function (1.75 ± 0.03 cm; mean ± *SEM*, *N* = 3,000), according to the pairwise comparison, and there was no significant difference between the constant (3.5 ± 0.04 cm, *N* = 3,000) and Gaussian functions (3.48 ± 0.02 cm, *N* = 3,000). However, the moving distance of the quadratic increase function (5.21 ± 0.03 cm, *N* = 3,000) was the highest (Figure 4).

## 4 DISCUSSION

In the present study, we showed that termites deposited most sand particles near the tip of the tunnel and characterized the distribution of sand deposition as a decrease quadratic function. Our result agreed with previous observations that *C. formosanus* excavates sand particles from the excavation site and deposits them to another site instead of pressing the sand particles into the tunnel (Li & Su, 2009).

Termite workers construct underground tunnels using their mouthparts and perform a four‐step tunneling behavior: (a) excavating sand, (b) loading sand into buccal cavities, (c) holding sand for transportation, and (d) deposition of sand (Li & Su, 2009). A termite is able to carry about 3.5 sand particles per trip on average, while depositing only one sand particle at time (Li & Su, 2009). In the tunnel branch formation of *C. formosanus*, the number of events of distal deposition was statistically higher than proximal deposition, except in the early stages of excavation (Bardunias & Su, 2009). The definitions of “proximal” and “distal” used in Bardunias and Su (2009) were based on the direction of tunnel branching. For instance, proximal deposition behavior was defined as when termites deposited sand particles toward the branching direction. If not, it was considered a distal deposition. In the present study, we were not able to detect this behavior described in Bardunias and Su (2009) since we were not focused on branching formation. However, we further showed that there was more amounts of deposited particles near the excavation point (1–2 cm from excavation site) than the farther away point (6–7 cm from excavation site).

Although termites were allowed to excavate the same distance, there were significantly more total amounts of deposited sand particles in the broadest tunnels with 4 mm of width. This is presumably due to high traffic efficiency. Wide tunnel widths positively contributed movement efficiency of *R. speratus kyushuensis* (Morimoto), a subterranean termite, showing that significantly less passing time required when two termites encountered in the tunnel (Sim & Lee, 2013). In addition to the traffic efficiency, termites were able to adjust tunnel widths to minimize stops in a wide tunnel (Lee, Bardunias, & Su, 2008c).

Other social insects such as ants also construct subterranean nests of interconnected chambers (Buhl, Gautrais, Deneubourg, Kuntz, & Theraulaz, 2006; Gravish et al., 2012; Monaenkova et al., 2015; Toffin, Paolo, Campo, Detrain, & Deneubourg, 2009; Toffin, Kindekens, & Deneubourg, 2010). Despite similarities in mechanisms of excavation with subterranean termites (i.e., excavation using mandibles), there is a fundamental difference in tunneling behaviors between ants and termites. Ants carried excavated particles to the surface (Gravish et al., 2012); however, there is no such surface for termites because they forage using underground galleries unless they reach food resources. In the case of ants, they must manipulate particles to maximize the amount soil per trip because they should carry excavated particles from the nest to the surface which is thousands of body length (Hooper‐Bui, Appel, & Rust, 2002; Monaenkova et al., 2015). Termites face the same situation in that they have to move back and forth to excavate, transport, and deposit. Unlike ants, if the moving distance between excavation and deposition site is long, the efficiency in tunnel construction for foraging decreased. From an ecological view point, we conjecture that the quadratic decrease distribution of deposited sand particles in both experiment and simulation is a strategy of subterranean termites to minimize energy expenditure in deposition behavior by sharing the work with more termites similar to food delivery in other social insects such as leaf cutting ants (i.e., bucket brigade; Ratnieks & Anderson, 1999).

Termites are classified by three different nesting habits, one‐piece (OP), intermediate, and separate‐piece (SP) types (Abe, 1987). OP termites (e.g., Kalotermitidae) inhabit a single piece of wood, which serves as both food and nest, while SP termites (e.g., Rhinotermitidae, Termitidae) have a central nest with multiple foraging sites (Abe, 1987). In addition to different nesting habits, colony size of SP termites is usually much bigger than OP termites. Subterranean termites are one of the SP termites which live as a colony underground and forage out by tunneling lengthy underground galleries. In the evolutionary transition between OP to SP termites, they may have evolved to increase efficiency in tunneling behaviors in order to minimize traffic jams and maximize foraging efficiency. Extra energy is needed in SP termites because they must bring food back to the central nest from the distant foraging site (Du, Chouvenc, Osbrink, & Su, 2017). In this process, excavation and deposition are key components. By minimizing energy expenditure in deposition, it is likely that termites may spend more energy in excavation and finding food resources to increase foraging efficiency.

The results of this study can be contributed to improve the agent‐based model (ABM) that can simulate termite tunneling behavior. One of the behavioral rules of simulated termites used in the ABMs is that simulated termites must picked up at the end of the tunnel and drop sand particles somewhere in the tunnel. Prior to this study, there were no experimental data to determine this distance. Applying the sand distribution function to the ABMs would give a more realistic simulated tunnel pattern closer to the actual termite tunnel pattern.

In conclusion, we found that deposited sand distribution in subterranean termites was quadratic decreased functions. Two steps of tunneling behaviors (i.e., excavation and deposition) are essential in subterranean termites to find food sources in distant area, while having high forging efficiency. The distribution of deposited particles could explain minimized energy expenditure in deposition behavior.

## CONFLICT OF INTEREST

The authors declare no conflict of interests.

## AUTHOR CONTRIBUTION

SBL and SHL designed the experiments. SBL conducted experiments. SHL did simulation. HSS analyzed image files. SBL, NYS, and SHL interpreted the result of experiments and the simulation. All authors contributed to the writing of the manuscript.

## DATA ACCESSIBILITY STATEMENT

All data produced from the current study will be deposited in Dryad after acceptance.
